# Comparison of Attenuation Imaging in the Rectus Femoris and Biceps Brachii Muscles with Multiecho Dixon-Based Fat Quantification and Ultrasound Echo Intensity

**DOI:** 10.3390/diagnostics15243239

**Published:** 2025-12-18

**Authors:** Sophia Zoller, Karolina Pawlus, Catherine Paverd, Thomas Frauenfelder, Florian A. Huber, Alexander Martin

**Affiliations:** 1 Faculty of Medicine, University of Zurich, 8032 Zurich, Switzerland; 2 Institute of Diagnostic and Interventional Radiology, University Hospital Zurich, 8091 Zurich, Switzerland

**Keywords:** echo intensity, attenuation imaging, ultrasonography, magnetic resonance imaging, skeletal muscle, musculoskeletal imaging, adipose tissue, sarcopenia, ultrasound, fat fraction

## Abstract

**Background/Objectives**: Sarcopenia, an underdiagnosed musculoskeletal disorder, is a serious cause of disability, poor quality of life, and healthcare costs in an increasingly elderly population. This study aimed to examine an ultrasound (US)-based, inexpensive, simple, and reproducible alternative to magnetic resonance imaging (MRI) for assessing muscle quality. A study compared Dixon MR fat fraction with US attenuation imaging (ATI) and echo intensity (EI) in the rectus femoris (RF) and biceps brachii (BB). **Methods**: The US images were acquired from 34 participants who had previously received a whole-body MRI. The ATI measurements were carried out using a linear array on a Canon Aplio i800 scanner. The measurements of EI were assessed by manually tracing the cross-sectional border of the right RF and BB muscles. Corresponding T1-weighted Dixon VIBE-based fat and water images were required for the MRI fat fraction percentage (MR %FF) measurements. **Results:** Using Pearsons correlation coefficient, a good correlation was found between MR %FF and EI measurements. The results between operators’ measurements showed a strong correlation and were highly repeatable. Attenuation imaging revealed no correlation with MR %FF or EI. **Conclusions**: Echo intensity offers a low-cost, non-invasive, and widely accessible US-based imaging modality for screening patients at risk for sarcopenia. No correlation was found between the ATI and MR %FF or between the ATI and EI. Further adapted protocols and software adjustments are needed so that ATI has the potential to prove itself as an additional US-based method for assessing fat infiltration in muscles.

## 1. Introduction

Musculoskeletal diseases and disorders are becoming more prominent, one of which is sarcopenia [1,2,3,4,5]. The current understanding of sarcopenia is based on the revised European consensus from the European Working Group on Sarcopenia in Older People (EWGSOP2) and is described as the generalised and progressive loss of skeletal muscle mass and low muscle strength [3,5,6]. Estimates of the size of the affected population range from 11% to 50% for those aged 80 or over and 5% to 13% for people aged 60 or older [7]. The aetiology is multifactorial and involves ageing, immobility, inadequate nutrition, or abnormal thyroid function [4,5]. Sarcopenia is associated with an increased risk of falls, frailty, and loss of independence and has been noted as a significant cause of morbidity and mortality in older adults [8,9,10]. This disorder remains underdiagnosed even though early intervention improves outcomes. As the number of elderly people rises, its clinical and economic significance will also increase [6,9,11,12].

Magnetic resonance imaging (MRI) is the preferred imaging modality for assessing muscle composition [13,14]. Among different quantitative techniques, Dixon MRI sequences are based on the chemical shift between water and fat signals [15,16]. In-phase and opposed-phase and, subsequently, fat and water images are created [15,16,17]. These images can be used to calculate the proton density fat fraction (PDFF), which is a meaningful quantitative imaging biomarker for fat quantification [18]. It describes the proportion of the density of protons from fat in the total density of protons from fat and water [19]. The clinical value of MRI lies in its high accuracy, reliability, and reproducibility, but is limited due to high costs and low availability [6,13].

Ultrasound (US) imaging is becoming routinely used for diagnosing sarcopenia due to its safety, as well as being inexpensive, non-invasive, and widely available clinically [6,17,20]. A range of US-based parameters, including muscle thickness, cross-sectional area (CSA), echo intensity (EI), and elastography, are available to evaluate the quantity and quality of muscle [9]. Ultrasound attenuation imaging (ATI) offers a promising avenue for exploration. ATI quantifies the frequency-dependent loss of ultrasound energy through tissue. ATI is widely used for hepatic steatosis assessment, where higher fat content increases attenuation [21,22,23,24]. ATI is routinely used to assess liver fat infiltration, specifically steatosis, and may therefore offer an early method for detecting changes in muscle quality. No study has previously used ATI and a clinically approved linear array to examine muscle quality.

Among US-based imaging techniques, echo intensity measurements, the mean grey-scale value within a region of interest on B-mode images, has been validated as a surrogate for intramuscular fat imaging, as adipocyte infiltration increases echogenicity [25,26,27,28,29]. Previous studies have demonstrated moderate to strong correlations between EI and MRI fat fraction in lower-limb muscles, particularly the rectus femoris (RF) [26,27]. EI has also shown utility in other muscle groups such as the multifidus [20], but its application to upper-limb muscles remains underexplored.

This study focuses on two anatomically and functionally distinct muscles: the rectus femoris (RF) and biceps brachii (BB). The RF is a large, superficial quadriceps muscle frequently examined in sarcopenia research due to its role in mobility and lower-limb strength [6,9,30]. Fat infiltration in the RF has been well documented, with MRI studies showing increased intramuscular fat in older adults and individuals with reduced physical activity [6,9,30]. EI has been shown to correlate strongly with MRI-derived fat fraction in the RF, making it a validated target for ultrasound-based assessment [26,27]. In contrast, the BB is a smaller upper-limb muscle critical for daily activities and independence. While ageing and neuromuscular disorders are associated with fat infiltration in the BB, quantitative data remain limited, and EI has not been extensively validated in this region [20,28]. Studying RF and BB together enables the evaluation of ultrasound-based techniques across different muscle sizes and anatomical regions, testing their reproducibility and clinical applicability. Although the cohort was largely non-sarcopenic, participants were older and at risk for sarcopenia, as reflected by median age (80 years) and SARC-F scores.

The primary objective of this study was to evaluate whether ultrasound-based parameters can serve as practical surrogates for MRI-derived fat fraction in clinically relevant muscles. To achieve this, we focused on the rectus femoris and biceps brachii, representing lower- and upper-limb muscle groups with distinct anatomical and functional roles. Our first aim was to quantify the association between echo intensity (EI) and magnetic resonance imaging fat fraction percentage (MR %FF) in these muscles, building on evidence that EI correlates with intramuscular fat in the thigh but remains underexplored in the upper limb [20,26,27,28]. Second, we assessed the feasibility and preliminary associations of attenuation imaging (ATI) with MR %FF using a clinically approved linear array, recognising ATI’s established role in hepatic steatosis yet its untested application in skeletal muscle. Finally, the inter-operator reliability of EI measurements in RF and BB was examined to determine reproducibility, a critical step for clinical translation [28].

## 2. Materials and Methods

### 2.1. Study Design

A prospective multicentre study design was used, in which each participant completed two testing sessions: a whole-body MRI assessment in the Balgrist Hospital or University Hospital of Zurich between 2020 and 2024 and an ultrasound assessment of the RF and the BB in the University Hospital of Zurich in April 2024.

Thirty-four study participants were selected (20 females and 14 males). The exclusion criteria included those whose images contained too many artefacts from their original MRI images. Participants’ age, sex, body mass index (BMI), excessive alcohol use (above 14 units of alcohol per week) [31], physical activity level, and sarcopenia score were assessed. These were obtained via a self-reported questionnaire. The sarcopenia score is determined using the SARC-F scale [32,33], which is a commonly used rapid diagnostic test for sarcopenia [6]. It consists of a 5-item questionnaire [33] and assesses muscle strength, assistance with walking, rising from a chair, climbing stairs, and falls [33]. A score of 4 or more is predictive of sarcopenia-related adverse outcomes [6,32,33]. Continuous variables are summarised below in Table 1 as median, [interquartile range], and (Range) when distributions were skewed, with ranges provided for context. Normality was assessed using the Shapiro–Wilk test. The local ethics committee (Kantonale Ethikkommission Zürich, KEK-ZH Nr. 2015-0323, 26 October 2015) and the institutional review board approved this multicentre study. Written informed consent was obtained from all participants before participating in the study. Previous studies have not published the volunteer data offered in this research. The study measurements and image segmentations were performed by two radiology residents with 1 and 3 years of experience, respectively. Both radiologists were given the same training and segmentation instructions from a senior board-certified consultant (FH) with 10 years’ experience.

### 2.2. Ultrasound Measurements

The TUS-AI800 US machine (Aplio i800, Canon Medical System Corporation, Otawara-shi, Japan) was used to perform an US assessment on the right side RF and BB muscles. A generous amount of US gel was used to ensure the best possible image quality and to avoid disturbing the image measurements by applying too much pressure to the skin.

The US settings, such as gain (76 dB), dynamic range (60), and time gain compensation (TGC), were kept consistent between the volunteers using a preset on the US machine and maintaining the TGC position to avoid accidental changes in these important parameters. The area of interest (AOI) was set to a depth of 10 cm, the ROI was set to a minimum of 2 × 2 cm, and the ROI was positioned so that the Canon system quality indicator, R^2^, was greater than 0.85.

For the rectus femoris and quadriceps femoris muscles, the patients were initially examined supine with legs extended and muscles fully relaxed. Rectus femoris scans were performed in both the transverse and longitudinal planes using a standard transducer position halfway between the anterior and superior iliac spine and the superior aspect of the patella. Due to limitations on the size of the region of interest (ROI) in ATI, the ROI included the rectus femoris and quadriceps femoris muscles.

For the biceps brachii, the patient was then instructed to sit upright, extend the arm forward, and then supinate while raising the arm slightly above the body. Images for BB muscle were acquired in the transverse plane, located two-thirds of the way between the acromion and the antecubital crease and used for assessment. This is due to its small longitudinal length and the difficulty of correctly positioning the US probe on the upper arm. The transducer was removed after each scan, and five measurements were taken consecutively for each muscle and each plane. Oblique scanning, which can lead to inaccurate muscle measurements, was avoided by placing the US probe at a 90° angle to the skin.

**Figure 1:**
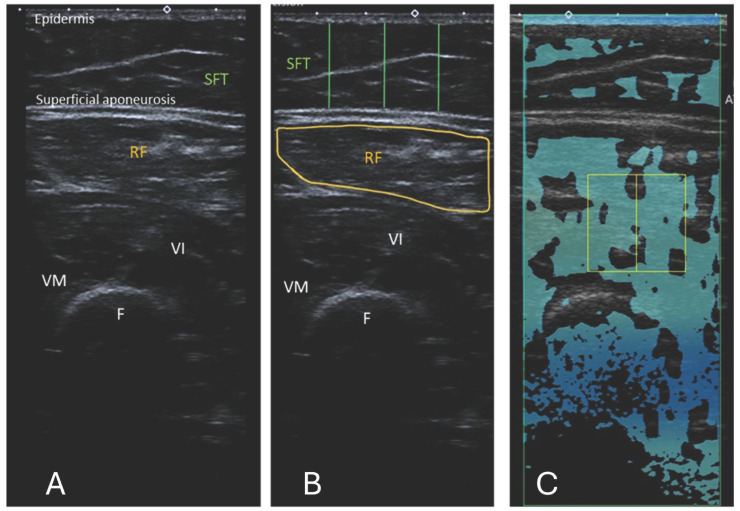
(**A**) Ultrasound B-mode image of the RF, musculus vastus intermedius, and medius. The distance between the epidermis and superficial aponeurosis is defined as subcutaneous fat tissue (SFT). (**B**) Segmented image of the RF, including the muscle cross-sectional area and the lines marking the thickness of the SFT from the lateral, central, and medial aspects of the RF. The measurement of the SFT was taken at 25%, 50%, and 75% of the total muscle visible on ultrasound, respectively. (**C**) Attenuation imaging of the RF. The green box indicates the area of interest (AOI), and the yellow box indicates the region of interest (ROI) placed within the quadriceps femoris muscles. RF = rectus femoris, VI = vastus intermedius, VM = vastus medialis, SFT = subcutaneous fat tissue, F = femur.

**Figure 2:**
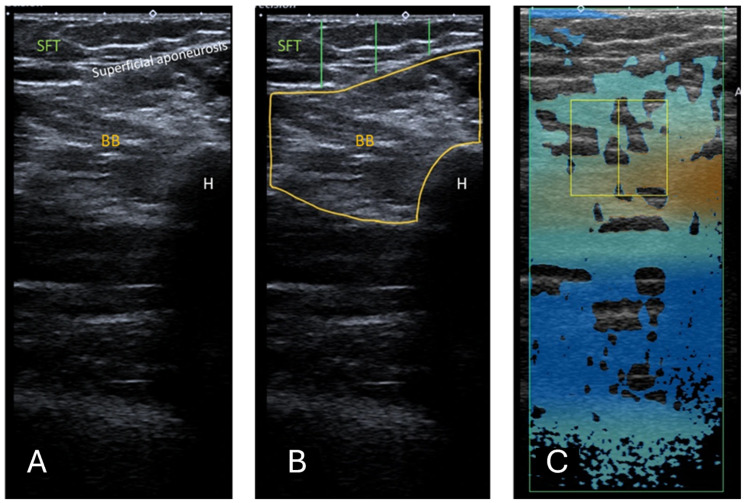
(**A**) Ultrasound B-mode image of the BB. The distance between the epidermis and superficial aponeurosis is defined as SFT. (**B**) Segmented image of the BB, including the muscle cross-sectional area and the lines marking the thickness of the SFT from the lateral, central, and medial aspects of the BB. The measurement of the SFT was taken at 25%, 50%, and 75% of the length of the total muscle visible on ultrasound, respectively. (**C**) Attenuation imaging of the BB. The green box indicates the area of interest (AOI), and the yellow box indicates the region of interest (ROI) placed within the BB. BB = biceps brachii, SFT = subcutaneous fat tissue, H = humerus.

10 US images were obtained for RF and 5 for BB (15 images per participant total), as shown in Figure 1 and Figure 2. The images were anonymised and saved as DICOM files for EI measurements.

#### 2.2.1. Echo Intensity

The images were processed using a computer-assisted greyscale analysis using ImageJ software (version 1.54h) [34]. Echo intensity is defined as the mean pixel intensity within the region of interest (ROI) and was calculated using the histogram function. The mean voxel intensity is expressed as a value ranging between black (0 arbitrary units) and white (255 arbitrary units). The RF and BB muscle cross-sections were circled manually to include as much of the muscle as possible, excluding any bone or surrounding myofascial tissue, as shown in Figure 1B and Figure 2B. A total of 5 consecutive images per level were taken, and the muscle was circled manually. The average of the 5 measurements was used in the analysis. When it was insufficient to display the entire muscle, only a part of the muscle was used for EI analysis. EI is taken only from transverse images for a fair and accurate comparison with ATI. The thickness of the subcutaneous fat tissue (SFT) was defined as the length from the epidermis to the superficial aponeurosis. For each transverse image, the SFT was drawn at 25%, 50%, and 75% of the total length of the BB or RF muscle visible on the ultrasound image, as shown in Figure 1B and Figure 2B. The average of these 3 lines was taken to have the distance for SFT. The average of the 5 measurements for SFT was used in the final analysis.

#### 2.2.2. Correction for Subcutaneous Fat Tissue

In accordance with the findings of previous studies, consideration was made for the confounding effect of the SFT on the EI [20,26]. Müller et al. [1] analysed the correlation between EI and an increasing adipose layer thickness by adding more exogenous layers of pork fat over the human tibialis anterior muscle. The formula with focus adjustment was used to calculate the correction factor (CF):

*EI corrected = EI measured + 39.2297* × *AT*

where

*EI corrected* is the *Echo intensity corrected value;*

*EI measured* is the *original echo intensity values* (without any corrections applied);

*AT* is the *adipose layer thickness.*

#### 2.2.3. Attenuation Imaging

The commercially available Canon linear array i11LX3 (Canon, Otawara-shi, Japan) with a centre frequency of 7 MHz was used to complete ATI of the RF and BB muscles. Attenuation imaging general (ATI-Gen) was the pre-set of the Aplio i800. The machine settings, such as a gain of 76 dB and dynamic range of 60, were kept consistent between participants. The depth of the image field was 10 cm when the area of interest (AOI) box was at maximum and as near to the middle of the frame as feasible, as shown in Figure 1C for RF and Figure 2C for BB. The ROI box was made to cover as small an area as possible (2 × 2 cm) and moved on the image so that R^2^ was greater than 0.85. Previous studies used an R^2^ value of 0.7 [24]; hence, 0.85 was deemed acceptable here. The box was positioned on the thigh to contain only the RF, where possible. This was not always possible due to the limitations in the smallest area of ROI available. In this case, the box was positioned on the RF, vastus intermedius, and medius muscles, including myofascial tissue. As the transverse cross-section of BB muscle is larger, the box can be positioned here so that no bone or muscle fascia lies on it as far as possible. Examples of the ATI measurement are shown in Figure 1 and Figure 2.

### 2.3. Magnetic Resonance Imaging Protocol

Whole-body MR images were acquired using a 3 Tesla MRI system (Magnetom Skyra, Siemens Healthineers, Erlangen, Germany) with a 36-channel coil. The Dixon VIBE sequence was performed with the following parameters: field of view (FOV) 68.75 cm; repetition time (TR) 4 ms; echo times (TE) 1.23/2.46 ms (in-phase/opposed-phase); flip angle 9°; bandwidth 1040 Hz/pixel; acceleration factor (GRAPPA) 2; breath-hold duration approximately 18–20 s per station; voxel size 1.8 × 1.8 × 3.0 mm; slice thickness 3 mm; number of slices per station 80; acquisition plane axial; and fat–water separation achieved using two-point Dixon reconstruction for fat and water images.

**Figure 3:**
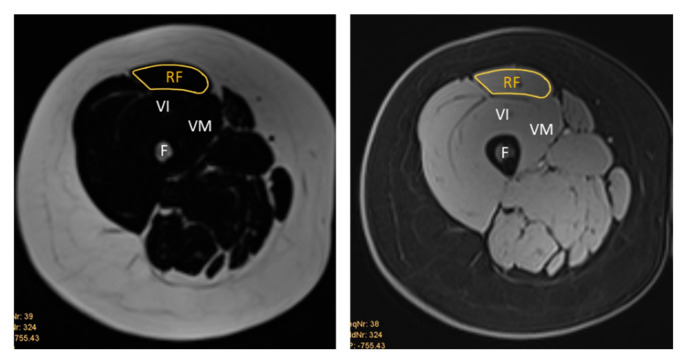
Representative T1-weighted Dixon VIBE MR image tracing the cross-sectional area of the right RF on the fat-based image (**left**) and water-based image (**right**). RF = rectus femoris, VI = vastus intermedius, VM = vastus medialis, F = femur.

**Figure 4:**
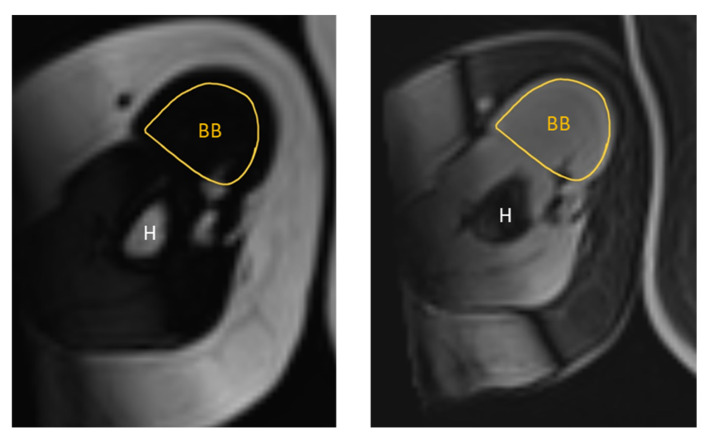
Representative T1-weighted Dixon VIBE MR image tracing the cross-sectional area of the right BB on the fat-based image (**left**) and water-based image (**right**). BB = biceps brachii, H = humerus.

The MR images were qualitatively analysed using the institution’s picture archiving and communication system (PACS). The same anatomical landmarks were used as for the US images. For the RF muscle, this was midway between the anterior and superior iliac spine and the superior aspect of the patella. For the BB muscle, the ROI was two-thirds of the way between the acromion and the antecubital crease. The MRI measurements were obtained by manually tracing the cross-sectional boundary of the right RF and right BB, as shown in Figure 3 and Figure 4. The boundary segmentation measurement was copied from the water image to the fat image, so the CSA had the exact same size. Using the axial water and fat images of the RF and BB muscles, the MR %FF was calculated using the following formula: MR %FF = (signal-fat/[signal-water + signal-fat] × 100) [27]. Each operator then segmented magnetic resonance images, which a senior doctor with more than ten years of experience then reviewed.

Young et al. [26] created a formula for calculating the estimated percentage of intramuscular fat based on SFT and EI. A comparison to measured values was made using the Young et al. calculation.

MR %FF: percent intramuscular fat = [0.093 × (40 × (subcutaneous fat thickness)) + rectus femoris echo intensity] + 4.698 [26].

A secondary analysis was conducted using another developed conversion question based on the corrected EI and age. The formula from Grozier et al. [27] is MRI percent intramuscular fat = −3.843 + (0.0.65 × corrected rectus femoris EI) + (0.145 × age). For the corrected EI, the mean EI and CF were multiplied as described by Müller et al. [1].

### 2.4. Statistical Analysis

Thirty-four participants were used in this study, in line with previous related investigations [20,26]. GraphPad Prism version 10.4.0 was used for the statistical analysis. A Pearson correlation coefficient was calculated for correlation matrices, and Bland–Altman plots were also produced using GraphPad Prism.

Inter-operator reliability for echo intensity (EI) measurements in the rectus femoris (RF) and biceps brachii (BB) was assessed using the intraclass correlation coefficient (ICC). A two-way random-effects, absolute-agreement model was applied to estimate reliability for single measurements (ICC(2,1)) and the average of both operators (ICC(2,2)). Model selection and reporting follow established guidance. Then, 95% confidence intervals were obtained via non-parametric bootstrap resampling of subjects (2000 iterations).

## 3. Results

**Figure 5:**
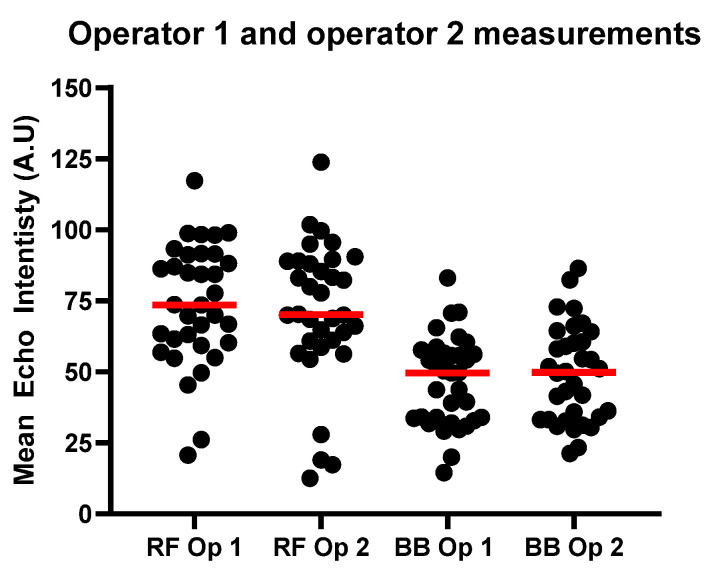
Results for RF and BB EI measurements for operators 1 and 2. The red line shows the median values for each group.

**Figure 6:**
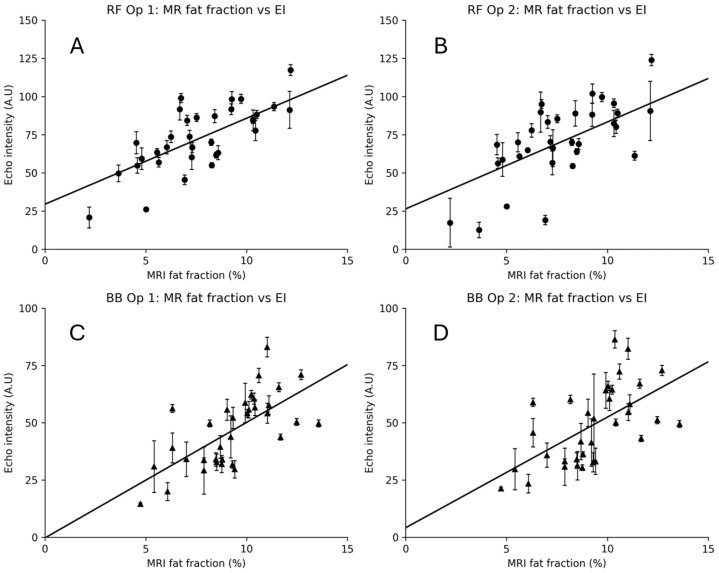
MRI fat fraction vs. echo intensity (EI) for rectus femoris (RF) and biceps brachii (BB). Panels show RF Op 1 (**A**), RF Op 2 (**B**), BB Op 1 (**C**), and BB Op 2 (**D**). Points represent participant-level means (five images per operator). Vertical error bars indicate within-participant EI SD. Circles used for RF measurements, triangles for BB measurements.

**Figure 7:**
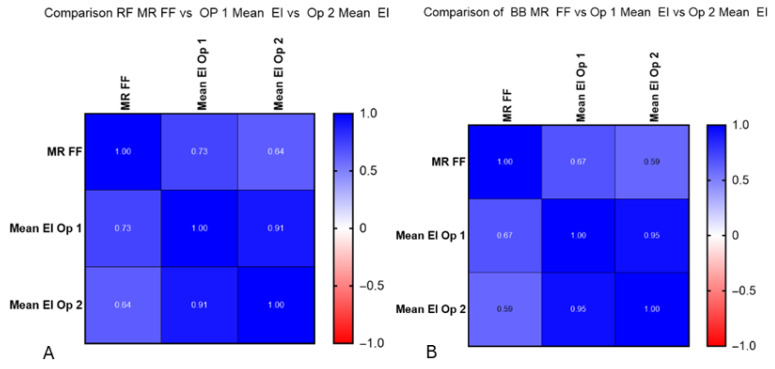
(**A**) Correlation between operators 1 and 2 and MR %FF calculations. Significantly strong correlations were found at RF for operator 1 (r = 0.73) and moderate at RF for operator 2 (r = 0.64). (**B**) Correlation between operators 1 and 2 and MR %FF calculations. Moderate correlations were found at BB for operator 1 (r = 0.67) and moderate at RF for operator 2 (r = 0.59).

**Figure 8:**
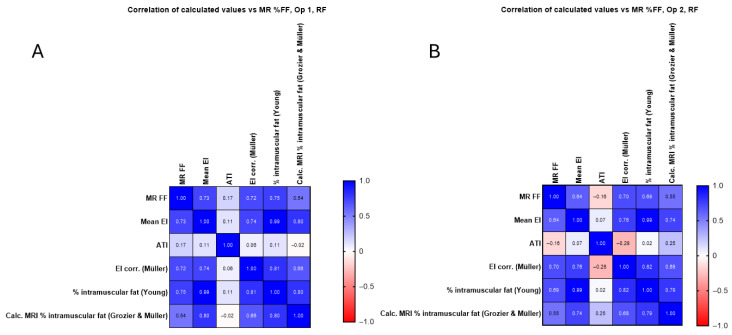
(**A**) Calculated corrected EI value and intramuscular fat calculations for operator 1 in the RF compared with original mean EI measurements and MR %FF, with no significant improvement seen. (**B**) Calculated corrected EI value and intramuscular fat calculations for operator 2 in the RF compared with original mean EI measurements and MR %FF.

**Figure 9:**
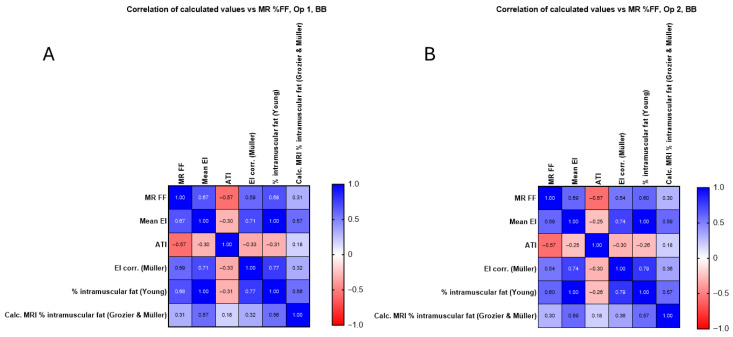
(**A**) Calculated corrected EI value and intramuscular fat calculations for operator 1 in the BB compared with original mean EI measurements and MR %FF, with no significant improvement seen. (**B**) Calculated corrected EI value and intramuscular fat calculations for operator 2 in the B compared with original mean EI measurements and MR %FF, with no significant improvement seen.

**Figure 10:**
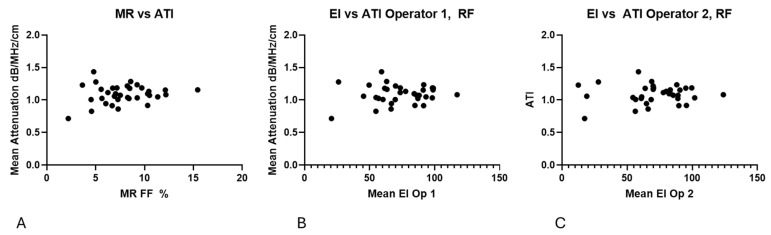
(**A**) No clear trend is present in the relationship between MR %FF and ATI measurements. (**B**) Echo intensity compared with ATI values, demonstrating no trend. (**C**) Echo intensity for operator 2 compared with ATI values, demonstrating no trend.

A comparison of EI, ATI, and MR %FF image analysis was initially made for each right RF and BB muscle and operators 1 and 2. The measurements are presented in Figure 5, Figure 6, Figure 7, Figure 8, Figure 9 and Figure 10, Table S1 for RF, and Table S2 for BB, in the Supplementary Materials.

### 3.1. Echo Intensity Measurements

Figure 5 is a comparison of the measurements conducted by both operators for mean EI from B-mode US images of the RF and the BB.

### 3.2. Echo Intensity Measurements Versus MR %FF

#### 3.2.1. Echo Intensity Measurements Versus MR %FF in the RF

Figure 6 shows the calculated MR %FF compared with the EI as measured by each operator. MRI fat fraction (MR %FF) values ranged from 2.19–15.44% in the rectus femoris (RF) and 4.73–13.58% in the biceps brachii (BB), with means of 7.87 ± 2.76% and 9.30 ± 2.09%, respectively. Echo intensity (EI) was higher in RF than BB, averaging 73.8 ± 21.3 A.U for RF Op 1 and 71.2 ± 24.7 A.U for RF Op 2, compared to 46.7 ± 15.6 A.U for BB Op 1 and 49.1 ± 16.9 A.U for BB Op 2.

EI demonstrated moderate-to-strong positive correlations with MR %FF across muscles and operators (Figure 6). For RF, the correlations were r = 0.73 (95% CI 0.52–0.86, *p* < 0.001) for Op 1 and r = 0.64 (95% CI 0.38–0.80, *p* < 0.001) for Op 2. For BB, EI correlated r = 0.67 (95% CI 0.43–0.82, *p* < 0.001) for Op 1 and r = 0.59 (95% CI 0.32–0.78, *p* < 0.001) for Op 2. The regression lines in Figure 7 illustrate these trends. Figure 7A shows the correlation for each operator and their EI results with MR %FF for the rectus femoris, and Figure 7B shows the same for the biceps brachii.

#### 3.2.2. Adjusted Echo Intensity Measurements Using Correction Methods and Intramuscular Fat Calculations

As stated in the Section 2, the original EI values obtained and shown in Figure 5, Figure 6 and Figure 7 were used with a correction factor to determine whether this would improve the correlation with MR %FF calculations. The results of those calculations can be seen in Tables S1 and S2 for operators 1 and 2, for both RF and BB measurements, found within the Supplementary Materials.

Figure 8 examines the correlation between the newly calculated corrected EI value and the intramuscular fat calculations using the methods described in the Materials and Methods. The relationship between each for RF is examined for each operator.

In Figure 8 and Figure 9, the corrected EI (Müller) and Young’s predicted intramuscular fat showed similar or slightly higher correlations in RF (up to r = 0.75) and BB (up to r = 0.68), whereas the Grozier and Müller combined estimates were weaker (r ≈ 0.30–0.54). The attenuation coefficient did not correlate significantly with MR FF in RF (r ≈ 0.17, *p* > 0.3) but showed a moderate negative association in BB (r ≈ −0.57, *p* < 0.001), consistent across operators.

The correlation between the calculated corrected EI value and the intramuscular fat calculations is shown with the values in Table S2. The relationship between each for BB is examined for each operator and is shown to not significantly improve when using additional methods.

As can be seen from Figure 8, there is no correlation between mean EI and ATI for RF (Figure 8A, operator 1 (r = 0.11) and operator 2 (r = 0.07)). As shown in Figure 9, there is a moderate negative correlation for operator 1 (r = −0.30, Figure 9A) and a weak correlation for operator 2 (r = −0.25, Figure 9B). There is no significant correlation between MR %FF and ATI at RF for operator 1 (r = 0.17, Figure 8A) and for operator 2 (r = −0.16, Figure 8B), as shown in Figure 8. Figure 9 shows a moderate negative correlation between MR %FF and ATI of BB for operator 1 (r = −0.57, Figure 9A) and operator 2 (r = −0.57, Figure 9B). The negative ATI–MR %FF trend in BB likely reflects anatomical constraints and ROI limitations rather than true physiology.

#### 3.2.3. Correlation Between Mean EI and ATI and MR %FF and ATI

No clear correlation could be found between EI, ATI and MR %FF, as shown in Figure 10.

## 4. Discussion

In this prospective study of 34 participants, echo intensity (EI) demonstrated moderate-to-strong correlations with MRI-derived fat fraction (MR %FF) in both the rectus femoris and biceps brachii, consistent with EI’s sensitivity to intramuscular adipose infiltration and connective tissue content [20,26,28,29]. The inter-operator agreement for EI was good to excellent by ICC, indicating robust repeatability of the ultrasound protocol across raters. In contrast, attenuation imaging (ATI) did not correlate with MR %FF and showed an unexpected negative trend in the biceps brachii, which we attribute to predefined ROI size constraints and local anatomy rather than true physiology [22,24]. Applying published conversion/correction formulae (Müller, Young, Grozier) did not materially improve agreement with MR %FF and, given their derivation in different muscle groups and cohorts, should be considered exploratory [1,26,27]. Collectively, these findings support EI as a promising, reproducible ultrasound marker of muscle quality, while highlighting that ATI requires protocol and software refinement before clinical translation.

EI increases with intramuscular fat and fibrosis because adipocytes and collagen elevate backscatter and brightness on B-mode ultrasound [28,29,35]. The observed EI–MR %FF associations therefore align with the expected biophysical mechanism of increased acoustic scattering in non-lean muscle. The stronger agreement and narrower limits of agreement in the biceps brachii likely reflect smaller inter-image variability and higher inter-operator reliability. In contrast, residual differences in the rectus femoris may be driven by heterogeneous echotexture and subcutaneous fat thickness [1,20,26].

Prior studies report moderate–strong EI–MRI fat correlations across lower-limb muscles, multifidus, and post-operative cohorts [20,26,27]. Our effect sizes are comparable to Young et al. (thigh/lower leg) and Grozier et al. (rectus femoris post-ACL reconstruction) [26,27]. By contrast, ATI has proven effective in hepatic steatosis where homogeneous parenchyma and larger ROIs yield stable attenuation coefficients [22,24]; the lack of association we observed in skeletal muscle and the negative ATI–MR %FF trend in the biceps brachii most likely stem from fixed ROI dimensions, fascia/bone adjacency, and multi-structure inclusion in the measurement box rather than a true inverse physiological relationship.

Inter-operator reliability was good–excellent (ICC(2,1) and ICC(2,2)), supporting the repeatability of EI segmentation and measurement. Bland–Altman analyses showed small biases and clinically acceptable limits of agreement, particularly in the biceps brachii. Differences in segmentation strategy between rectus femoris and biceps brachii were necessitated by anatomy (smaller cross-section, proximity to humerus and fascia) and are acknowledged as potential sources of systematic measurement differences. The scanner’s minimum ROI size constrained ATI measurements.

Previous studies have examined the confounding effect of SFT on the evaluation of EI [1,20,26,27]. The mean EI value decreases when the thickness of the SFT increases [1]. In their study, Müller et al. [1] analysed the correlation between EI and an increasing adipose layer thickness by adding more exogenous layers of pork fat over the human tibialis anterior muscle. However, adding correctional methods does not significantly improve the measured values. As shown in Figure 8A, the correlation between the corrected EI values and MR %FF is about 0.59, which is lower than 0.67 for mean EI and MR %FF. The same trend is seen for operator 2 when examining the BB (0.54, 0.59).

The results presented here correspond to the study results of Crook et al. [20], that there is a mixed improvement of mean EI vs. corrected EI [26]. The confounding effect of SFT was assessed using external pressure on the skin with the US probe. As the skin is compressed, the tissue density and, therefore, the EI values vary. As Müller et al. [1] demonstrate, this method does not allow the quantification of the independent effect of the SFT layer. For the purpose of this study, the most recently published and adapted formula of Müller et al. [1] was used. Despite the use of the most recently adapted formula, there was a considerable lack of improvement between the measured EI value and the mentioned CF for the confounding effect of SFT.

A limitation of this study is the application of previously published correction formulas (Müller et al. [1], Young et al. [26], Grozier et al. [27]) to muscle groups beyond those for which they were originally validated. These formulas were developed for specific anatomical regions and experimental conditions, and their direct transferability to the biceps brachii may be limited. Differences in muscle architecture, fibre orientation, and surrounding tissue could influence echo intensity and attenuation characteristics, reducing the accuracy of these predictive models. Future work should focus on developing and validating muscle-specific correction factors for upper limb muscles. Further studies are required to consider the confounding effect of fat on estimated EI values.

Attenuation imaging (ATI) has demonstrated strong clinical utility in hepatic steatosis, where higher fat content consistently increases attenuation coefficients, enabling the reliable quantification of liver fat infiltration [21,22]. However, its application to skeletal muscle remains unexplored, and our findings highlight important challenges for translation. In this study, ATI showed no significant correlation with MRI-derived fat fraction in the rectus femoris (RF) and an unexpected moderate negative correlation in the biceps brachii (BB). This contrasts sharply with the positive association observed in hepatic tissue and suggests that current ATI protocols may not be directly transferable to musculoskeletal imaging. We attribute these discrepancies primarily to technical limitations rather than true physiological differences. Specifically, the predefined minimum ROI size on the Aplio i800 scanner (2 × 2 cm) often exceeded the cross-sectional area of the RF, forcing the inclusion of adjacent structures such as fascia and neighbouring muscles, resulting in measurements of the quadriceps femoris muscles. This likely introduced heterogeneity in attenuation measurements and masked any real association with intramuscular fat. In the BB, the negative trend may reflect compounded effects of small muscle size, proximity to bone, and anisotropic tissue architecture, which influence ultrasound beam propagation and attenuation. These findings underscore that ATI, while promising in homogeneous organs such as the liver, requires substantial protocol refinement, including adaptive ROI sizing and muscle-specific calibration, before it can be considered a viable tool for skeletal muscle assessment.

The principal limitations are the MRI-ultrasound time interval, the modest sample size from a strictly defined cohort (prior whole-body MRI), differences in anatomy-driven segmentation, and constraints on the ATI ROI. The published formulae (Müller, Young, and Grozier) were not designed for the biceps brachii and did not generalise in our data [1,26,27].

Future work should (i) perform same-day MRI and ultrasound to eliminate interval bias [18]; (ii) develop muscle-specific EI/ATI protocols and calibration curves for upper-limb muscles; (iii) evaluate smaller, adaptive ATI ROIs that reliably exclude fascia and bone; (iv) undertake multicentre validation with larger cohorts; and (v) assess home/handheld translation alongside clinic-based systems [17].

In summary, EI showed reproducible associations with MR %FF across two anatomically distinct muscles, whereas ATI, under current constraints, did not. These findings support EI as a supplementary tool for muscle quality assessment and emphasise the need for protocol and software refinement before attenuation-based methods can be reliably applied to skeletal muscle.

The supplementary data examines the links between BMI and fat infiltration. There is no clear correlation between BMI and MR %FF within the RF or the BB. However, an important consideration must be made. The BMI values includes the whole body and not just a single muscle group. There are differences in the fat distribution between individuals. The BMI shows no correlation with either the mean EI or MR %FF results presented within this paper. There is still no clear identifiable correlation between an increase in BMI and an increase in the mean EI. This follows the same trend as MR %FF, which is unsurprising given that the measurement area for mean EI and the MR %FF is the same muscle group.

## 5. Limitations

This study has several limitations. First, the modest sample size (*n* = 34) and the wide age range reflect strict inclusion criteria requiring prior whole-body MRI, which limits generalisability. Second, the time interval between MRI and ultrasound assessments introduces potential bias, although most participants were clinically stable and non-sarcopenic. Third, attenuation imaging was constrained by the scanner’s minimum ROI size (2 × 2 cm), which often encompassed adjacent quadriceps femoris structures rather than isolating the rectus femoris. This likely diluted attenuation measurements and contributed to the lack of correlation with MRI fat fraction. Fourth, segmentation strategies differed between muscles due to anatomical constraints, which may have introduced systematic variability. Fifth, previously published correction formulas (Müller, Young, and Grozier) were applied to muscle groups beyond their original validation context, limiting their accuracy for the biceps brachii. Finally, the exploratory nature of this study precludes definitive diagnostic thresholds; future work should include same-day MRI and ultrasound, larger cohorts, and muscle-specific calibration protocols.

## 6. Conclusions

Echo intensity (EI) demonstrated reproducible, moderate-to-strong correlations with MRI-derived fat fraction in both the rectus femoris and biceps brachii, supporting its potential as a low-cost, accessible adjunct for assessing muscle quality. Inter-operator reliability was high, reinforcing EI’s clinical feasibility. In contrast, attenuation imaging (ATI) showed no meaningful association with MRI fat fraction and an unexpected negative trend in the biceps brachii, likely driven by technical and anatomical constraints rather than true physiology. These findings underscore that ATI, while effective in homogeneous organs such as the liver, requires substantial protocol refinement, including adaptive ROI sizing and muscle-specific calibration, before translation to skeletal muscle imaging. Future research should prioritise same-day MRI and ultrasound acquisition, the development of muscle-specific correction factors, and validation in larger, diverse cohorts to enable robust clinical adoption.

## Figures and Tables

**Table 1:** Participants’ characteristics. The median values, interquartile range [IQR], and the range of values are given in parentheses.

	Median [IQR] (Range)
Age (years)	80.2 [76.2–82.1] (21.1–88.3)
Sex	20 female, 14 male
Weight (kg)	71.6 [60.3–81.1] (51–120)
Height (m)	1.66 [1.60–1.74] (1.45–1.90)
Body Mass Index (kg/m^2^)	24.01 [22.78–29.07] (20.4–40.6)
Sarcopenia score	1 [0–1] (0–5)

## Data Availability

The data from this study is available on request. The data are not publicly available due to privacy and hospital policy.

## Supplementary Materials

The following supporting information can be downloaded at https://www.mdpi.com/article/10.3390/diagnostics15243239/s1, Table S1. Showing the mean calculated EI values for operators 1 and 2 for the BB, and the corrected values for EI, the calculated intramuscular fat percentages using Young et al. [26], and Grozier et al. [27], methodologies. Table S2. Showing the mean calculated EI values for operators 1 and 2 for the BB, and the corrected values for EI, the calculated intramuscular fat percentages using Young et al. [26], and Grozier et al. [27], methodologies.

## Abbreviations

AOI	Area of interest
ATI	Attenuation imaging
ATI-Gen	Attenuation imaging general
BB	Biceps brachii
BMI	Body mass index
CF	Correction factor
CSA	Cross-sectional area
EI	Echo intensity
EWGSOP2	Revised European consensus from the European Working Group on Sarcopenia in Older People
MRI	Magnetic resonance imaging
MSK	Musculoskeletal
PDFF	Proton density fat fraction
RF	Rectus femoris
ROI	Region of interest
SFT	Subcutaneous fat tissue
US	Ultrasonography
MR %FF	Magnetic resonance imaging fat fraction percentage
