# Supplementary material for: Comparison of Attenuation Imaging in the Rectus Femoris and Biceps Brachii Muscles with Multiecho Dixon-Based Fat Quantification and Ultrasound Echo Intensity
Source: Diagnostics (Basel). 2025 Dec 18;15(24):3239. doi: 10.3390/diagnostics15243239 (PMC12731617; doi:10.3390/diagnostics15243239)
Supplement: Supplementary file 1 [file diagnostics-15-03239-s001.zip › diagnostics-4008109-supplementary.pdf]

Table S1. Showing the mean calculated EI values for operators 1 and 2 for the BB, and the corrected values for EI, the calculated intramuscular fat percentages using Young et al.[22], and Grozier et al.[23], methodologies.

RF Operator 1			
Mean EI OP 1	EI corr. (Müller)	% intramuscular fat (Young)	Calc. MRI % intramuscular fat (Grozier & Müller)
20.71	35.22	26.79	9.47
49.62	105.47	59.62	14.47
45.41	99.61	55.25	14.09
91.57	191.14	105.71	20.18
91.60	135.75	100.48	16.73
63.16	112.77	72.56	15.67
56.90	123.01	67.87	16.77
59.23	87.92	66.65	14.05
54.84	93.12	63.17	14.39
93.35	132.69	101.78	17.54
73.58	127.82	83.42	16.36
73.63	104.93	81.29	14.87
77.73	96.76	84.23	14.48
66.56	94.75	73.93	13.92
69.71	98.74	77.16	14.03
66.81	89.06	73.62	14.42
84.34	139.64	94.28	16.69
84.33	138.76	94.19	16.78
60.27	105.87	69.29	14.93
63.40	83.34	69.99	13.32
91.22	138.60	100.41	17.20
87.08	100.59	93.06	14.15
98.21	149.19	107.74	17.45
98.97	132.59	106.85	16.38
98.41	139.04	106.96	16.94
86.23	126.18	94.72	16.54
26.14	99.92	37.83	5.70
54.98	143.61	68.08	13.18
98.80	200.05	113.10	20.04
88.13	186.39	102.15	14.36
61.59	109.40	70.82	11.39
69.95	110.54	78.50	11.61
84.91	187.09	99.29	14.70
117.27	202.72	130.07	19.19

RF Op 2			
Mean EI	EI corr. (Müller)	% intramuscular fat (Young)	Calc. MRI % intramuscular fat (Grozier & Müller)
17.34	31.85	23.41	9.25
12.54	68.38	22.53	12.06
19.02	73.22	28.85	12.37
89.67	189.24	103.81	20.06
88.08	132.24	96.97	16.50
68.84	118.45	78.24	16.04
60.85	126.95	71.82	17.02
58.70	87.39	66.12	14.02
56.26	94.54	64.59	14.48
61.18	100.53	69.61	15.45
77.79	132.03	87.63	16.63
70.29	101.60	77.96	14.65
80.04	99.06	86.54	14.63
66.09	94.28	73.46	13.89
68.45	97.49	75.91	13.95
64.85	87.10	71.66	14.29
82.28	137.58	92.23	16.55
83.27	137.70	93.13	16.71
56.50	102.10	65.53	14.68
70.01	89.96	76.60	13.75
90.51	137.89	99.70	17.15
88.92	102.42	94.90	14.27
101.85	152.83	111.38	17.69
95.02	128.64	102.91	16.12
99.63	140.26	108.18	17.02
85.36	125.31	93.84	16.48
27.95	101.73	39.64	5.81
54.42	143.05	67.53	13.14
83.13	184.38	97.43	19.02
89.03	187.29	103.05	14.42
63.89	111.70	73.12	11.54
69.99	110.58	78.53	11.61
95.56	197.75	109.95	15.39
123.87	209.33	136.68	19.62

Table S2. Showing the mean calculated EI values for operators 1 and 2 for the BB, and the corrected values for EI, the calculated intramuscular fat percentages using Young et al.[22], and Grozier et al.[23], methodologies.

BB Operator 1			
Mean EI	EI corr. (Müller)	% intramuscular fat (Young)	Calc. MRI % intramuscular fat (Grozier & Müller)
39.01	49.38	44.69	10.39
29.71	44.88	35.85	10.53
19.98	46.10	27.16	10.61
43.85	68.67	50.90	12.22
58.64	82.37	65.59	13.26
70.64	90.51	77.23	14.22
33.93	68.46	41.90	13.22
31.98	48.85	38.28	11.51
32.79	60.08	40.08	12.24
60.49	86.27	67.63	14.52
31.78	48.76	38.09	11.22
52.16	75.54	59.07	12.96
39.43	70.47	47.07	12.77
57.72	67.42	63.33	12.14
33.96	51.85	40.36	10.98
56.64	64.03	62.04	12.79
55.70	75.28	62.26	12.51
55.71	76.67	62.39	12.74
34.08	55.27	40.79	11.64
30.87	46.71	37.07	10.94
54.12	74.28	60.73	13.02
29.16	53.00	36.12	11.06
83.05	116.45	90.92	15.33
49.72	72.61	56.58	12.48
56.21	76.86	62.86	12.90
62.23	94.33	69.97	14.47
14.51	56.78	23.21	2.89
33.66	122.40	46.77	11.80
50.36	95.75	59.36	13.26
65.54	97.55	73.28	8.59
43.74	74.69	51.37	9.13
54.17	88.30	62.10	10.16
49.69	97.31	58.90	8.86
71.02	100.54	78.52	12.55

BB Operator 2			
Mean EI	EI corr. (Müller)	% intramuscular fat (Young)	Calc. MRI % intramuscular fat (Grozier & Crook)
45.65	56.03	51.34	11.03
33.19	48.35	39.32	11.07
23.41	49.52	30.58	11.37
41.42	66.24	48.47	12.57
64.17	87.89	71.11	14.10
72.35	92.21	78.93	14.74
36.24	70.77	44.21	14.08
30.42	47.28	36.71	11.76
31.23	58.52	38.52	12.70
86.42	112.19	93.56	16.74
32.71	49.69	39.02	11.63
51.81	75.19	58.72	13.42
41.80	72.84	49.44	13.57
58.13	67.84	63.75	12.37
34.03	51.92	40.42	11.36
50.18	57.57	55.58	12.52
60.55	80.13	67.10	13.22
54.32	75.28	61.01	13.08
35.86	57.04	42.56	12.19
29.75	45.59	35.95	11.19
54.65	74.81	61.26	13.47
30.83	54.67	37.79	11.66
82.35	115.75	90.22	15.97
60.16	83.06	67.03	13.63
58.93	79.59	65.59	13.50
21.26	63.53	29.97	3.33
33.18	121.92	46.29	11.77
51.17	96.56	60.17	13.31
67.08	99.09	74.82	8.69
43.15	74.10	50.78	9.09
66.11	100.24	74.05	10.94
49.54	97.16	58.75	8.85
72.88	102.40	80.38	12.67
